# Orchestrated regulation of iron trafficking proteins in the kidney during iron overload facilitates systemic iron retention

**DOI:** 10.1371/journal.pone.0204471

**Published:** 2018-10-15

**Authors:** Avital Weiss, Lior Spektor, Lyora A. Cohen, Lena Lifshitz, Inbar Magid Gold, De-Liang Zhang, Marianna Truman-Rosentsvit, Yael Leichtmann-Bardoogo, Abraham Nyska, Sefi Addadi, Tracey A. Rouault, Esther G. Meyron-Holtz

**Affiliations:** 1 Laboratory for Molecular Nutrition, Faculty of Biotechnology and Food Engineering, Technion-Israel Institute of Technology, Haifa, Israel; 2 Molecular Medicine Program, Eunice Kennedy Shriver National Institute of Child Health and Human Development, National Institutes of Health, Bethesda, Maryland, United States of America; 3 Sackler School of Medicine, Tel Aviv University, and Consultant in Toxicologic Pathology, Timrat, Israel; 4 B-nano Limited, Rehovot, Israel; CINVESTAV-IPN, MEXICO

## Abstract

The exact route of iron through the kidney and its regulation during iron overload are not completely elucidated. Under physiologic conditions, non-transferrin and transferrin bound iron passes the glomerular filter and is reabsorbed through kidney epithelial cells, so that hardly any iron is found in the urine. To study the route of iron reabsorption through the kidney, we analyzed the location and regulation of iron metabolism related proteins in kidneys of mice with iron overload, elicited by iron dextran injections. Transferrin Receptor 1 was decreased as expected, following iron overload. In contrast, the multi-ligand hetero-dimeric receptor-complex megalin/cubilin, which also mediates the internalization of transferrin, was highly up-regulated. Moreover, with increasing iron, intracellular ferritin distribution shifted in renal epithelium from an apical location to a punctate distribution throughout the epithelial cells. In addition, in contrast to many other tissues, the iron exporter ferroportin was not reduced by iron overload in the kidney. Iron accumulated mainly in interstitial macrophages, and more prominently in the medulla than in the cortex. This suggests that despite the reduction of Transferrin Receptor 1, alternative pathways may effectively mediate re-absorption of iron that cycles through the kidney during parenterally induced iron-overload. The most iron consuming process of the body, erythropoiesis, is regulated by the renal erythropoietin producing cells in kidney interstitium. We propose, that the efficient re-absorption of iron by the kidney, also during iron overload enables these cells to sense systemic iron and regulate its usage based on the systemic iron state.

## Introduction

The kidneys are extremely sensitive to heme and hemoglobin exposure during hemolytic anemias [1–4]. In contrast, they are rarely mentioned amongst tissues that are damaged by elemental iron overload such as the liver, spleen, heart and pancreas [5–7]. In recent years it has become clear that significant amounts of plasma transferrin (Tf) pass through the glomerular filter and reach the primary urine [8–10]. In Fanconi syndrome, glomerular filtration is normal, but proximal tubule (PT) re-absorption is impaired and much Tf is found in the urine of these patients [11]. This supports the notion that Tf is passing through the glomerular sieve and reaches the primary urine also under physiological conditions, in which hardly any iron or protein is secreted through urine and thus Tf and its bound iron must be reabsorbed [12]. Tf bound iron is taken up by the renal epithelium through receptor-mediated endocytosis. The two main mediators for Tf reabsorption are cubilin and Tf Receptor (TfR)1, both proteins are found apically in the PT epithelium [13–15]. In mice, TfR1 was detected not only on the PT, but also on apical membranes of collecting ducts [10]. It was also found in distal convoluted tubules of rats [16] and in all tubular epithelia of humans [17].

Most iron transport proteins including TfR1 are regulated by iron to maintain cellular and systemic iron homeostasis. Iron uptake by TfR1 is post-transcriptionally down-regulated by the Iron Regulatory Protein (IRP)-system when the cellular iron is high [18]. The two IRPs, IRP1 and IRP2 bind to the stem-loop structures on TfR1 mRNAs and stabilize the short-lived TfR1 transcript when cellular iron is low. In contrast, when cellular iron levels are high, TfR1 expression and cellular iron uptake are low, which is one of the mechanisms that protect cells from iron overload. Nevertheless, efficient iron trafficking occurs across the kidney epithelium, even when the systemic iron is high.

Another promising candidate receptor that permits renal Tf and iron reabsorption during iron overload is the multi-ligand receptor, cubilin [19]. Cubilin is a 460 kDa protein that is located in many epithelial barriers, including the apical PT-membrane [20]. It does not have a transmembrane domain and thus cannot anchor to the plasma membrane independently. Cubilin binds to the multi-ligand receptor megalin for membrane-localization and internalization, and to the protein amnionless mainly for cellular trafficking [15]. The megalin-cubilin complex is the major multi-specific receptor complex in the kidney, which is responsible for the re-absorption of many proteins, nutrients and other compounds filtered through the glomerulus, including Tf [15].

Non-transferrin bound iron (NTBI) may be bound to albumin which is also taken up through the megalin-cubilin complex or it is bound to small molecules such as citrate and is taken up through their transporters or is reduced and reabsorbed as ferrous iron (Fe^2+^) [21–23]. Fe^2+^ is transported by the divalent metal transporter 1 (DMT1), that is expressed apically in the Loop of Henle [24] or through one of the ZIP transporters. However, the relative importance of this route is not known. In the cortical PT and collecting ducts most DMT1 has an intracellular, endo/lysosomal location [25]. Thus, DMT1 collaborates there with Tf-internalizing receptors as a transporter of iron from the endo/lysosomal system to the cytosol, following iron-release from Tf after internalization.

Once iron is absorbed into the epithelial cells, it is either used for cellular needs or basolaterally exported. Intermittently iron is stored in the iron storage protein ferritin which is also regulated by the IRPs but in the opposite direction of TfR1, as IRP binding to the two ferritin-subunit transcripts in low iron conditions inhibits ferritin translation. Thus, ferritin is highly expressed during iron overload, as well as in mice with targeted deletion of IRP2 [26].

Ferroportin (FPN) is the only ferrous iron exporter known to date and is located basolaterally on kidney epithelium, where it mediates the exit of iron from the epithelial cell and thus completes re-absorption [27]. FPN is regulated on many levels, including transcriptionally by HIF, translationally by IRPs and at the level of protein function and degradation by the peptide hormone hepcidin. Hepcidin regulates iron homeostasis systemically as it blocks iron export by arresting the conformation transition of FPN necessary for transport [28, 29] and induces FPN internalization and breakdown [30, 31]. This reduces dietary iron uptake by intestinal epithelial cells and iron efflux by many cells, including macrophages after erythrophagocytosis and hepatocytes that function as long-term iron stores. Other iron export pathways such as ferritin- or heme-secretion may play a role in iron trafficking through kidney epithelium [32, 33]. In physiologic conditions, ferritin localizes near the apical membrane of the tubule in the polarized kidney epithelium [32, 34].

In this research we studied proteins involved in iron re-absorption through the kidney during Parenterally induced systemic Iron Overload (PIO). PIO was elicited by iron dextran-injections, that mimic parenteral administration of iron supplements. We found that TfR1 is indeed down-regulated. In contrast, cubilin is highly up-regulated during PIO. We further show evidence that the cubilin-regulation by iron is post-transcriptional and may be mediated by an iron-dependent stabilization of cubilin through the transcriptional up-regulation of megalin. In epithelial cells, the intracellular ferritin distribution is shifted towards the basolateral membrane, close to the interstitium. In the interstitium, iron and ferritin accumulate mainly in macrophages, and more prominently in the medulla than in the cortex. This stands in contrast to iron absorbed through the diet, where iron accumulates mainly in the renal epithelium.

## Materials and methods

### Animals and PIO

Animal experiments were done according to protocols approved by the Technion Animal Ethics Committee, Haifa, Israel. All mice were on C57Bl/6J background. PIO was elicited in 3 month-old male mice by 5 daily intra-peritoneal injections of 100μl iron-dextran solution (90mg iron/ml, Sigma-Aldrich). Three days after the last injection mice were sacrificed, kidneys were collected, frozen in liquid nitrogen and stored at -80°C.

### Histological methods

Kidneys and spleens were collected and fixed in 10% neutral buffered formalin or 4% PFA, respectively. The kidneys were trimmed mid-longitudinally, to include the cortex, medulla and papilla, and then embedded in paraffin, sectioned to a thickness of approximately 5 microns and stained with Hematoxylin & Eosin (H&E) for histology and Perl’s Prussian blue (PPB) for ferric iron detection. Histopathological changes were analyzed and scored using a semi-quantitative grading of five grades (0–4), taking into consideration the severity of the changes (0 = no lesion, 1 = minimal change, 2 = mild change, 3 = moderate change, 4 = marked change). Spleens were prepared and analyzed similarly.

### Immunohistochemistry (IHC) and Immunofluorescence (IF)

Paraffin-embedded kidney sections from iron overloaded and control mice were heated in the microwave for 10 minutes for antigen retrieval with 6M urea in PBS (for all proteins) or with 0.01M citrate buffer (for H-ferritin) and rinsed with ddH_2_O. For IHC, quenching of endogenous peroxidase was performed by incubating the slides in 3% H_2_O_2_ in a humid chamber following by ddH_2_O washes. Next, sections were blocked with 10% normal goat/donkey serum (Jackson) in PBS containing 0.1% bovine serum albumin (BSA, Sigma) that was chosen according to the animal in which the secondary antibody was raised. Then, sections were incubated overnight (ON) at room temperature (RT) in a humidified chamber with the following primary antibodies; polyclonal goat-anti-mouse cubilin (diluted 1:200, Santa Cruz Biotechnology), monoclonal mouse-anti-human TfR1 (diluted 1:200, Zymed), polyclonal affinity purified rabbit-anti-mouse H- or L-ferritin (a kind gift from Prof. A. M. Konijn, Hebrew University, Jerusalem; diluted 1:200) and polyclonal goat-anti-mouse CD68 (diluted 1:200, Santa Cruz Biotechnology). For IHC staining, sections were washed with PBS and incubated for 1 hour at RT with biotinylated goat-anti-rabbit or rabbit-anti-goat antibodies (diluted 1:500, Vector). Then, sections were incubated with Vectastain ABC kit following manufacturer's instructions. The immunohistochemical reaction was visualized using DAB (Sigma). Sections were dehydrated and mounted with Eukitt resin (Sigma). For IF staining, donkey-anti-goat 488, donkey-anti-rabbit 568 and goat-anti-mouse 488 were used as secondary antibodies and incubated for 1 hour at RT (diluted 1:1000, Invitrogen). Next, slides were washed with PBS and mounted with VECTASHIELD mounting medium containing DAPI (Vector laboratories). Negative controls were incubated with secondary antibodies only.

### Ferric iron stain

Paraffin-embedded kidney sections from iron overloaded and control mice were blocked with peroxidase blocking solution (3% H_2_O_2_ in PBS), stained with PPB solution (2% K_2_Fe(CN)_6_, 2% HCL mixed freshly in a 1:1 ratio) for 1 h and washed several times with ddH_2_O. Positive staining appeared as a blue color under light microscope.

### Quantitative PCR (qPCR)

Total tissue RNA was isolated from kidneys using Trizol reagent (Invitrogen). Samples were treated with DNase I recombinant, RNase free kit (Roche) according to the manufacturer's instructions. cDNA was synthesized using total RNA (1 μg) by qScript cDNA synthesis kit (Quanta biosciences), and was amplified using SYBER Green (Quanta biosciences) in AB 7300 (Thermo Fisher Scientific).

Primers:

*TfR1* sense 5' TGG GTC TAA GTC TAC AGT GGC 3' and anti-sense 5' AGA TAC ATA GGG CGA CAG GAA 3';

*Cubilin* sense 5' AGG CTG TGG AGG CAA TCT CA 3' and anti-sense 5' GGT AGT AGG GCA TCG GGT AGT 3';

*Megalin* sense 5' AGG CCA CCA GTT CAC TTG CT 3' and anti-sense 5' AGG ACA CGC CCA TTC TCT TG 3';

*β-actin* sense 5' AGC CTT CCT TCT TGG GTA TGG 3' and anti-sense 5' TCA ACG TCA CAC TTC ATG ATG G 3'.

### Correlative microscopy *with air*-SEM and EDX detector

Paraffin-embedded kidney sections were fixed on Superfrost microscope slides. High iron kidney slides were H&E stained and left uncovered for *air*SEM (B-nano LTD) analysis. First, slides were imaged with an optical microscope for sample orientation and selection of region of interest, images were acquired using 20X, 40X and 100X objectives, then the sample was moved automatically to the SEM, where matching fields were imaged using backscattered electron imaging as described.[35, 36] Elemental analysis and mapping were carried out by an EDX detector placed on the same optical axes of the SEM.

### Immunoblotting

Proteins separated on SDS-PAGE were transferred to a nitrocellulose membrane. For blocking, membranes were incubated in 5% BSA for 1 h at RT (cubilin, actin and TfR1), in 3% BSA, ON, at 4°C for ferritin, and in 2% BSA and 3% milk ON, at 4°C for FPN. Next, the membranes were incubated ON, at 4°C, with the following first antibodies: Cubilin (diluted 1:500, A20 Santa Cruz), actin (diluted 1:5000, Santa Cruz), TfR1 (diluted 1:2000, Invitrogen), while the incubation with anti-ferritin antibodies was for 1 h at RT (diluted 1:5000, a kind gift from Prop. A. M. Konijn) and with anti-FPN antibodies was for 3 h at RT (1:500, produced in Rouault lab). HRP-conjugated anti-goat (diluted 1:10,000, Sigma) or anti-rabbit (diluted 1:25,000, Jackson) antibodies were used as secondary antibodies for analysis of cubilin/actin and TfR1/ferritin/FPN, following 1 hr incubation at RT.

## Results

### No morphological kidney damage due to parenteral systemic iron overload

**Fig 1:**
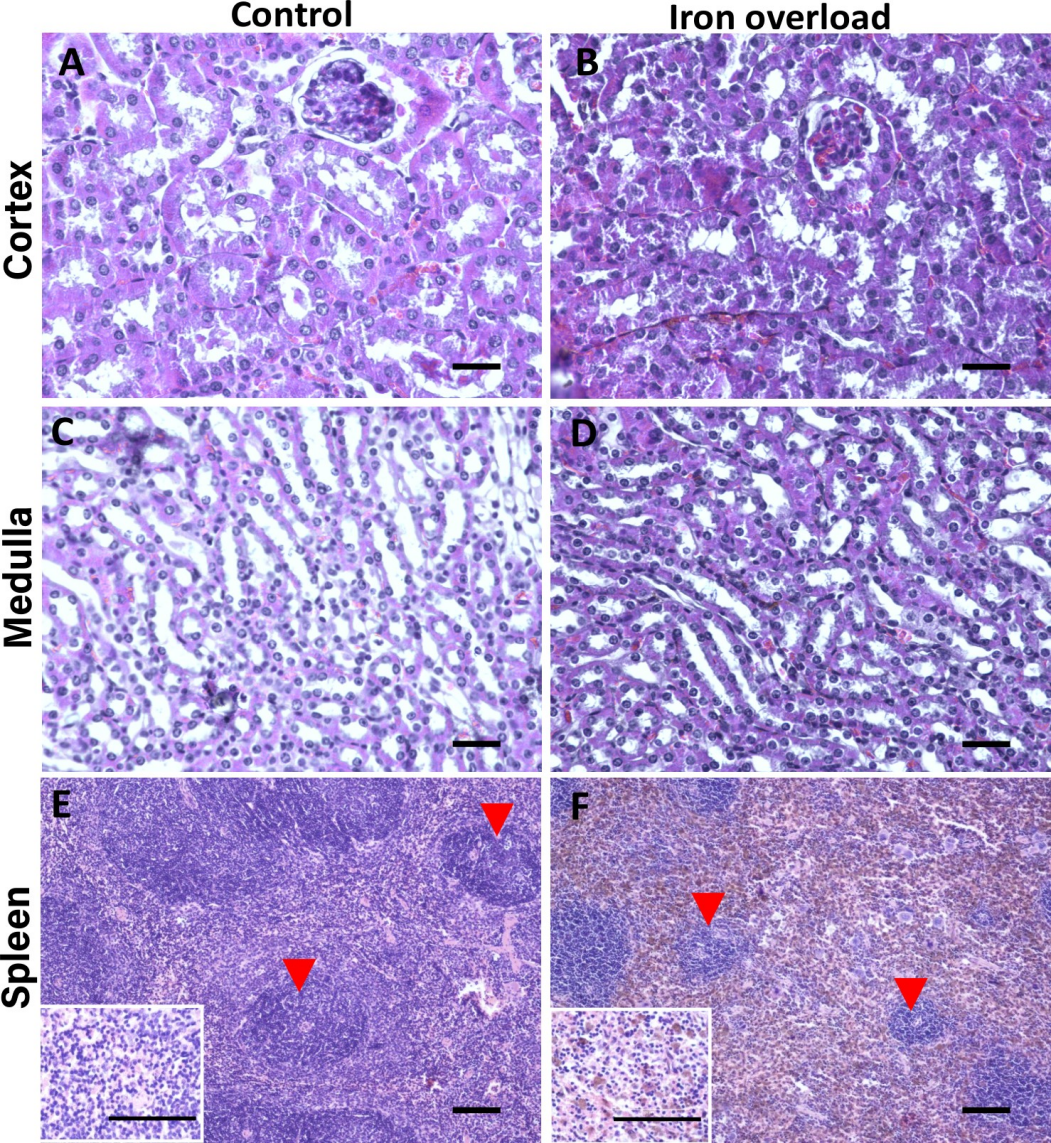
PIO did not cause morphologic kidney damage, but affected the spleen. Fixed kidney and spleen sections from control and iron-loaded mice were histologically stained (H&E) and imaged. No damage was observed in kidney sections of iron-loaded mice **(B and D)** compared to the control sections **(A and C)**. However, iron accumulation caused damage to the spleen as can be evaluated by moderate degree of hemosiderosis (seen as brownish pigment in the red pulp of section **F** compared to **E**), and by a mild depletion of lymphocytes in the white pulp (indicated by red arrowheads); the inserts in the left corner of E and F are higher magnifications of these sections. Scale bar 100 μm, n = 3 for control and iron-loaded mice each.

To study how iron transport is regulated during PIO, we caused PIO in mice by injecting iron dextran into the peritoneum. Histological analysis of kidney and spleen sections of these mice showed completely normal kidney morphology with well-defined glomeruli and tubules. In the kidney, no iron accumulation can be detected in H&E staining, without specific visualization of iron (Fig 1A–1D). In contrast, in the spleens of the same mice we detected hemosiderosis (brown haze, Fig 1F), a mild depletion of lymphocytes in the periarteriolar lymphoid sheath of the white pulp, and the boundaries of the white pulp were not well defined (Fig 1E and 1F).

### Cubilin is found in the medulla of iron-overloaded mice

**Fig 2:**
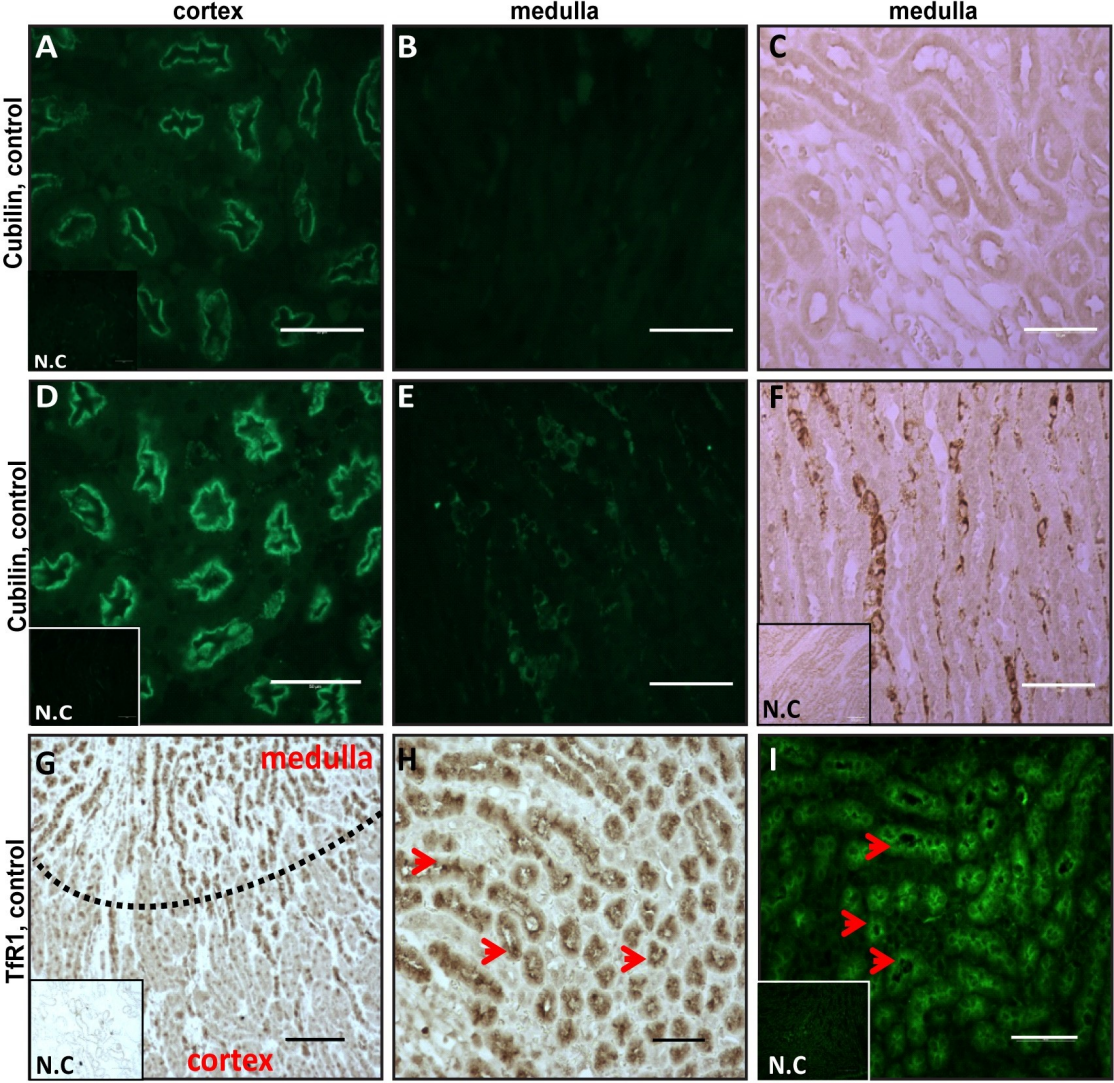
Cubilin expression in iron loaded kidneys is elevated both in the cortex and the medulla. The fixed kidney sections were incubated with cubilin or TfR1 antibodies and stained by IF or IHC. **(A-F)** Cubilin was observed apically in the cortex, as expected, with increased staining in kidneys from iron overloaded mice (compare **A** to **D**). Interestingly, in PIO, significant cubilin expression was also observed in the medulla (compare **B** to **E**, and **C** to **F**). Inserts are negative controls (N.C) for cubilin staining; **(G-I)** Visualization of TfR1 by IHC **(G-H)** and by IF **(I)**. **(G)** TfR1 staining was seen mainly in the medulla, (the dashed line separates the cortex from the medulla. In the medulla, TfR1 was seen apically **(H-I)** (indicated by arrows). Inserts are negative controls (N.C) for TfR1 staining; scale bar of 50μm, n = 3 for control and iron-loaded mice each.

**Fig 3:**
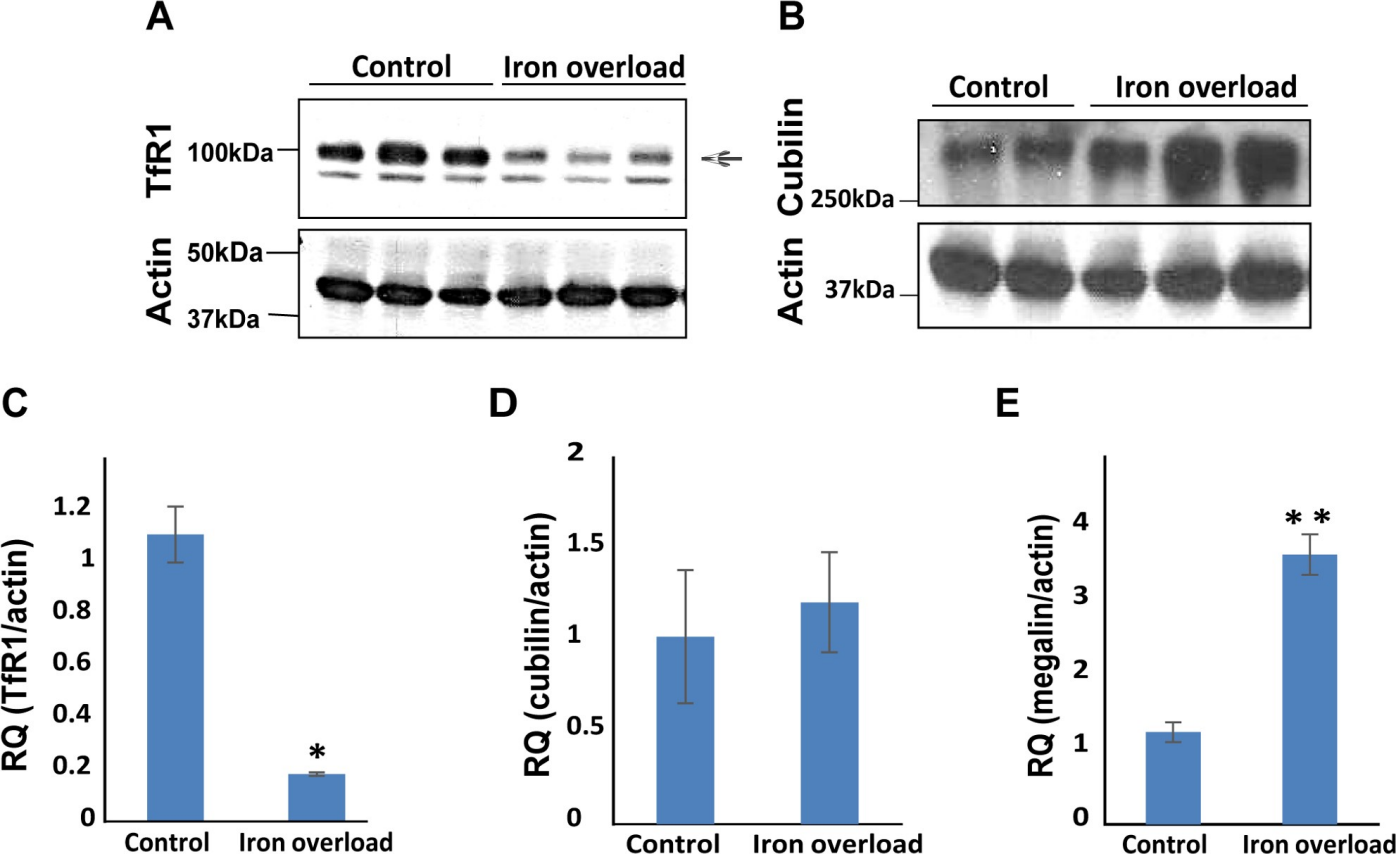
Cubilin and Tfr1 are regulated by iron in opposite directions. TfR1 (**A**) and cubilin (**B**) protein levels were tested by Western-blot analysis of kidney lysates. Membranes were probed with either cubilin, TfR1 or actin antibodies. TfR1 levels in iron over-loaded mice were decreased. Cubilin levels were increased in kidneys from iron–loaded mice. **(C, D and E)** Kidney mRNA levels of TfR1, cubilin and megalin were evaluated by qPCR. **(C)** TfR1 mRNA levels correlated with protein levels **(**n = 5, *P<0.05), **(D)** no significant difference in cubilin mRNA levels was observed **(**n = 6). **(E)** Megalin mRNA expression increased in iron overloaded kidneys (n = 7, **p<0.005).

To understand how Tf-iron uptake systems are regulated in the kidney during iron-overload, we studied the expression of these systems, which are known to be expressed at the apical epithelial membrane. Both cubilin and TfR1 play a role in Tf and iron uptake to the epithelium and thus we localized both candidates (Fig 2). At the same time we planned to use cubilin as a marker for the apical membrane of the cortex as described [37]. As expected, we detected cubilin expressed apically in the cortex, but surprisingly, in iron-overloaded mice, it was not only highly upregulated in the cortex, it was also detectable in the medulla (Fig 2A–2F). This suggested an iron mediated up-regulation of cubilin. In control mice, TfR1 was expressed in the cortex as described earlier [10], but was also found in the medulla, further supporting that the medulla is playing an important role in the re-absorption of iron from the primary urine. In the kidney sections of iron overloaded mice, TfR1 was under detection limits, consistent with the instability of TfR1 mRNA during iron overload (Fig 3A and 3C).

### Cubilin and TfR1 are regulated in opposite directions by PIO

As expected, TfR1 protein and mRNA levels were decreased in the kidneys of iron overloaded mice (Fig 3A and 3C, respectively). In contrast, cubilin-protein was highly up-regulated in the kidneys of these mice (Fig 3B), supporting the observation of the immunofluorescence experiment (Fig 2A–2F). The cubilin protein elevation could not be explained by a transcriptional regulation of cubilin, as mRNA levels were not elevated in PIO mice (Fig 3D).

### Megalin mRNA is elevated in kidneys of iron overloaded mice

To further investigate the elevated cubilin levels in kidneys of iron overloaded mice we wondered if megalin, that is part of the megalin-cubilin complex, is affected by iron as well. Indeed, megalin mRNA expression was highly elevated in the kidneys of iron overloaded mice (Fig 3E), suggesting a role for megalin in the iron mediated upregulation of cubilin.

### Epithelial ferritin undergoes an iron-mediated intracellular redistribution

**Fig 4:**
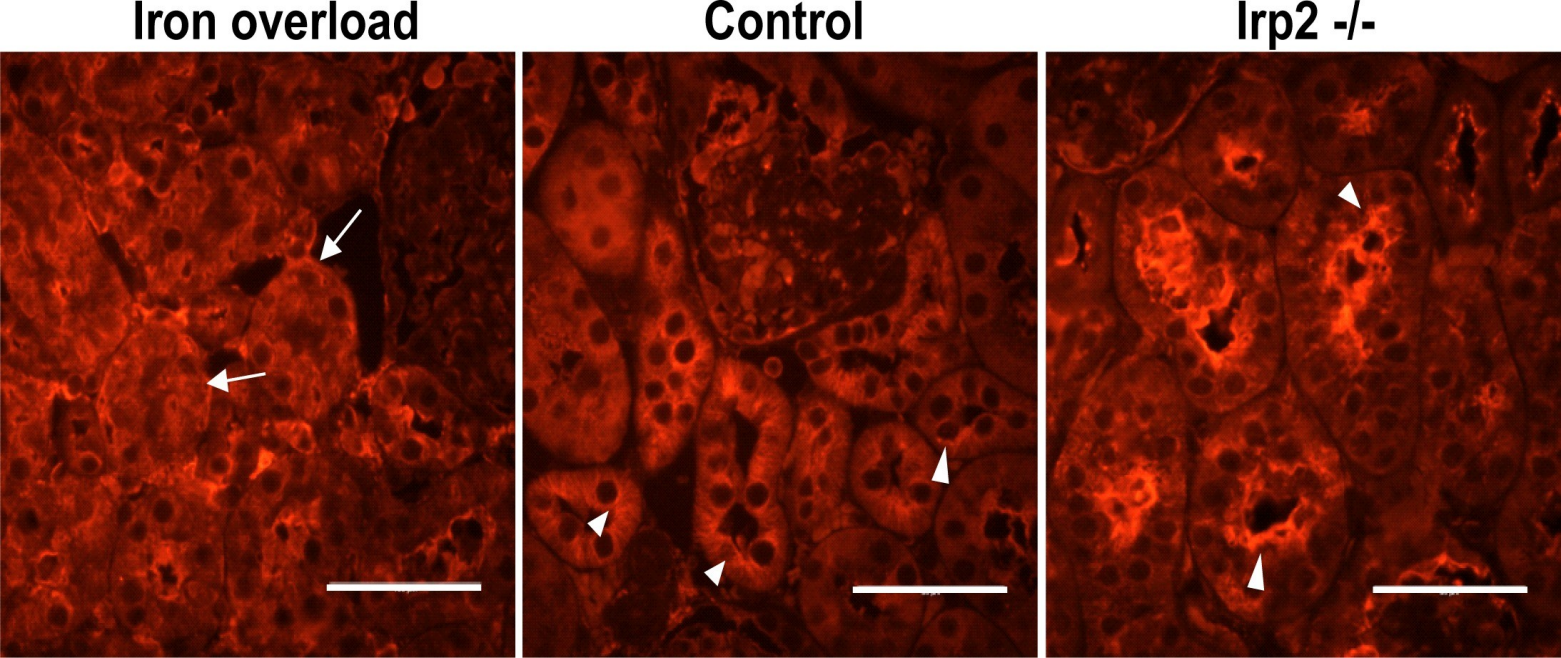
Ferritin distribution in renal epithelial cells is regulated by the iron status: Kidney sections from control, iron-loaded and Irp2-/- mice were stained with H-ferritin antibody. Ferritin was apically polarized in kidneys from Irp2-/- mice and to some extent also in wild-type control mice (arrow heads). In contrast, in iron overloaded mice ferritin was distributed throughout the cells and also found in basolateral regions (arrows). Scale bar represents 50μm.

In renal epithelial cells of control mice, ferritin has a polarized appearance near the apical brush-border of the PT [32, 34]. We noticed that in the PT epithelium of iron overloaded mice, this polarization was completely lost and ferritin was distributed throughout the epithelial cells (Fig 4). This suggested that cellular iron level might regulate intracellular ferritin distribution. To test this hypothesis, we analyzed epithelial localization of ferritin in Irp2-/- mice, which suffer from a functional iron deficiency [38–40]. Indeed, in the Irp2-/- mice ferritin was strongly polarized in the apical pole of the epithelial kidney cells (Fig 4), further supporting a regulated ferritin distribution within this polarized cell-type, with an apical enrichment of ferritin in cells with lower iron concentration.

### Iron export from renal epithelial cells

**Fig 5:**
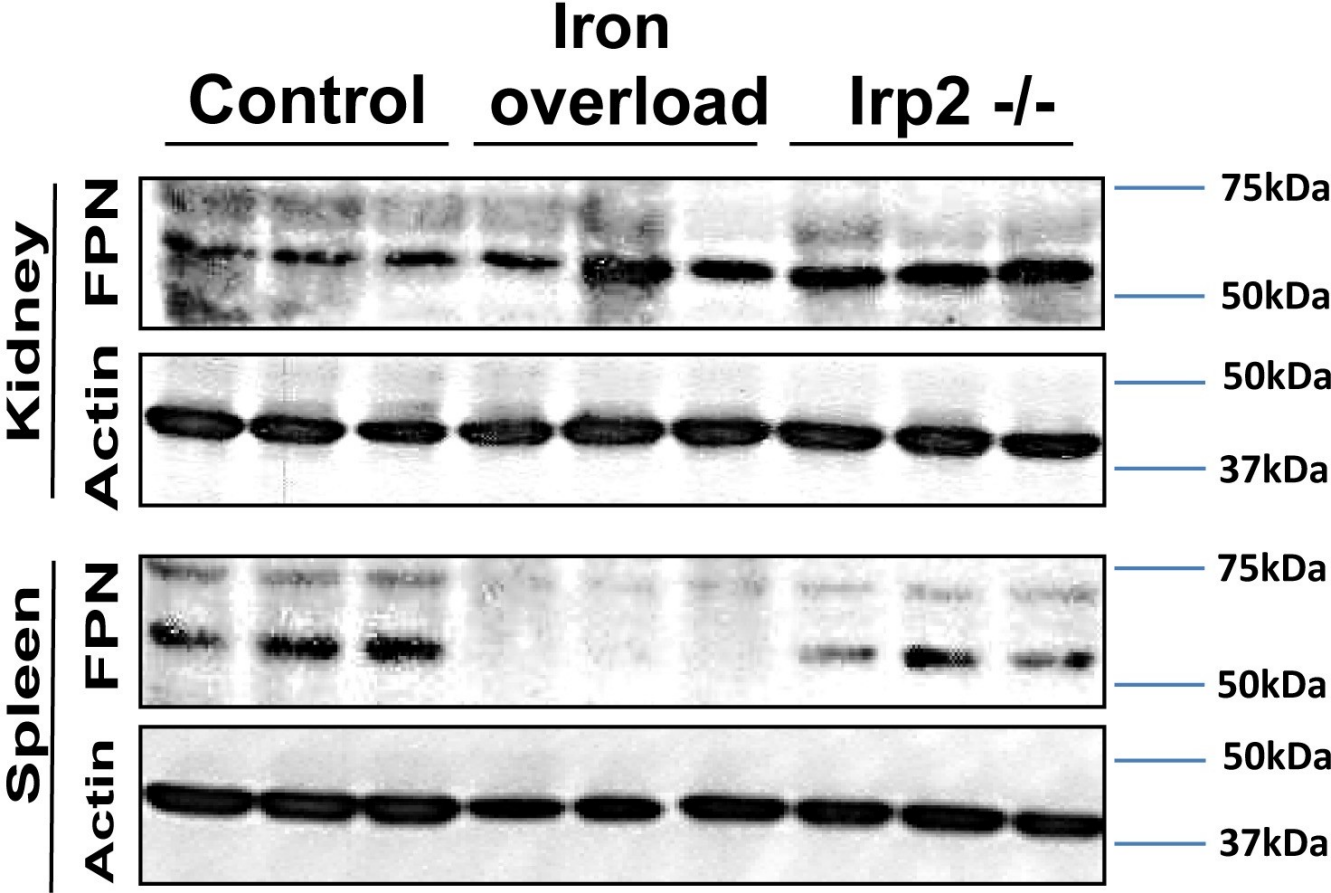
FPN levels are not reduced in kidneys of iron overloaded mice. Spleens and kidneys were lysed in 1% triton buffer and 40μg protein was submitted to Western blot analysis following protein separation by 10% SDS-PAGE. Proteins were electro-transferred to a nitrocellulose membrane, which was incubated with antibodies against FPN or actin. FPN was detected as a 60kDa band.

To further study the route of iron through kidney epithelium, we asked whether the ferrous iron exporter FPN was regulated also in the kidney by the iron status of the mouse. We found that FPN was not reduced by PIO in the kidney, but was significantly reduced in the spleen of the same mice (Fig 5). In mice with a targeted deletion of IRP2, spleen FPN was slightly reduced and FPN in the kidney slightly elevated. These data suggested that in the kidney, iron is exported efficiently from cells that express FPN also during PIO.

### Parenterally administered iron accumulates in interstitial macrophages, mainly in the medulla

**Fig 6:**
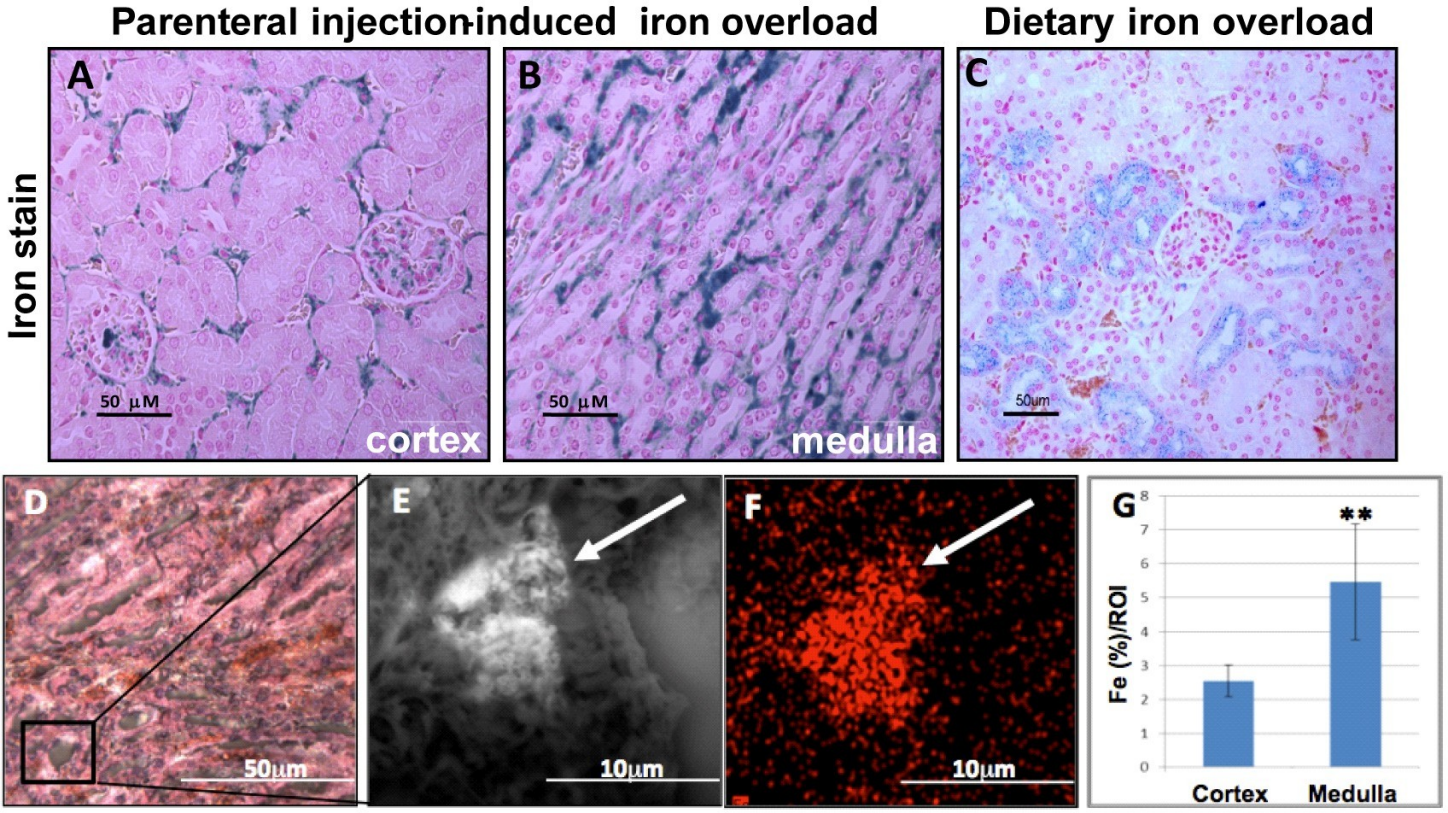
Iron accumulates in the kidney-interstitium of PIO mice. Fixed kidney sections from PIO mice (A-B and D-F) and from dietary iron overloaded mice (C) were stained and imaged. **(A-C)** Ferric iron was visualized with Prussian blue staining in cortex and medulla, respectively. In PIO mice, most iron accumulated in and around glomeruli and in the interstitium, and markedly little iron was detected in renal epithelial cells. In contrast, following dietary iron overload, most iron accumulated in proximal tubule epithelium of the cortex. **(D)** Light microscope; the black square indicates a sub-region of the medulla, which is enlarged in (E and F). **(E)** AirSEM analysis of the sub-region followed by EDX [Fe] mapping **(F)** indicated iron accumulation (marked with arrows) in the interstitium of the medulla. **(G)** Regions of interest in tubules and interstitium of cortex and medulla were selected and iron levels were quantified using airSEM. At least 2-fold increase of interstitial iron in the medulla compared to cortex was measured. ** P< 0.0001.

**Fig 7:**
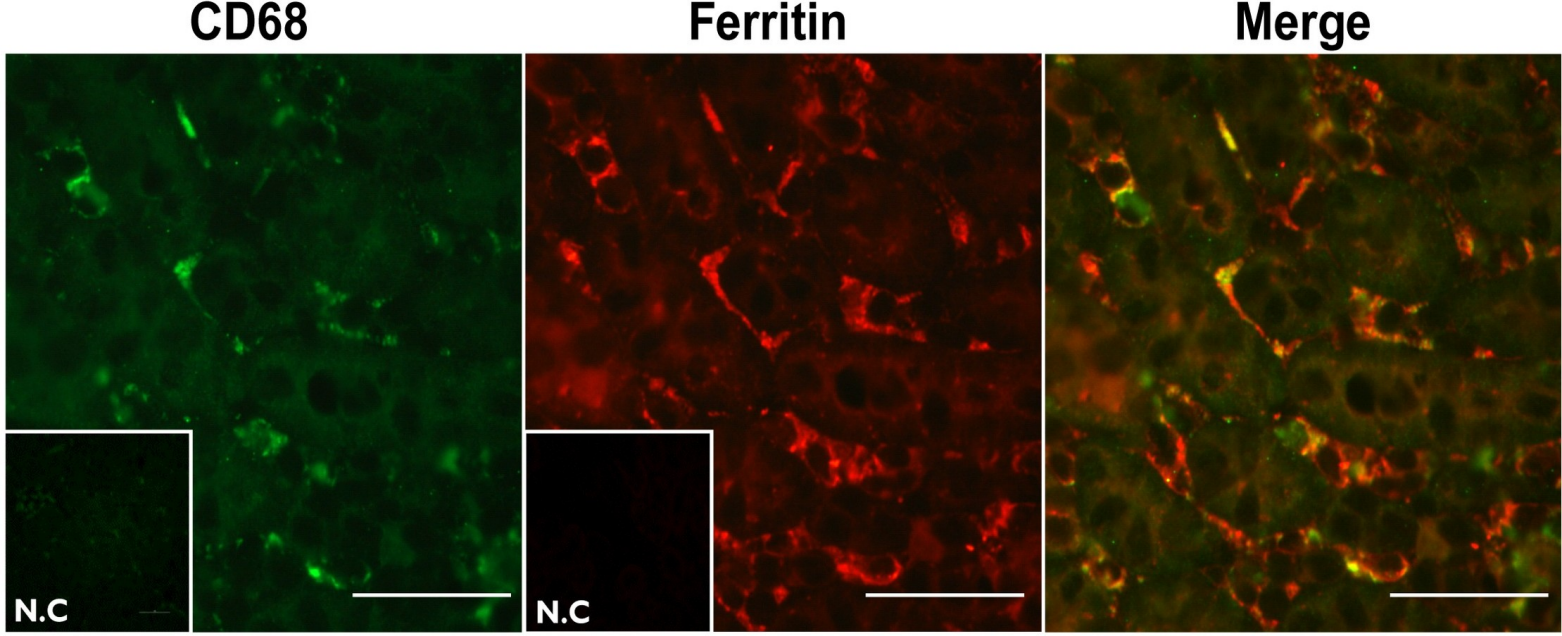
Ferritin accumulated in medullary interstitial macrophages: Kidney sections from PIO mice were co-stained with anti-CD-68 and L-ferritin antibodies. Co-localization can be observed as yellow regions in the right panel. Scale bar represents 50μm.

To test where iron accumulates in the kidney during iron overload, we compared iron accumulation in kidneys from parenterally and dietary iron-overloaded mice. Iron was first visualized by PPB staining and was significantly enriched in the medullar interstitium of PIO mice (Fig 6A). In these mice, iron was also detected in the cortical interstitium near the PT and in and near the glomerulus, but not near the distal tubules (Fig 6A). The epithelial cells were spared from iron overload (Fig 6A and 6B). Interestingly, following dietary iron overload, iron accumulated mainly in the proximal tubule epithelial cells of the cortex (Fig 6C), whereas there was little staining in medulla, suggesting that different mechanisms are behind the handling of dietary or parental administration of iron overload. In order to better characterize and quantify iron content per compartment, correlative microscopy (epi-fluorescence and SEM) was used to image both optical-, and backscattered electron-images by *air*SEM and metal content was analyzed by EDX (Fig 6D–6F). This verified the iron accumulation in the medullar interstitium and quantification of the interstitial areas in cortex and medulla showed two times more iron in the medullar than in the cortical interstitium (Fig 6G). To identify the cell-type in which iron accumulates, we co-stained kidneys from iron-overloaded mice with ferritin and the macrophage-marker CD68. PPB stain visualizes ferric iron (Fe^3+^), which is mainly found in ferritin and hemosiderin. Indeed, ferritin immune-staining presented a very similar pattern to PPB stain and localized predominantly to the interstitium in the kidneys of PIO mice. Most of the ferritin co-localized with the CD68-macrophage-marker (Fig 7) indicating that iron is mostly accumulated in interstitial macrophages.

## Discussion

Several regulatory mechanisms protect the mammalian organisms from iron overload, including control of iron uptake, recycling and storage by hepcidin, the IRPs, hypoxia-inducible factor (HIF) and NCOA4 [41–45]. In contrast to most cell types, where this regulation inhibits cellular iron uptake during iron overload, we show that kidney epithelial cells have all the tools to re-absorb most iron from primary urine regardless of systemic iron status. Thus, it can be assumed that a considerable amount of iron traffics through the kidney during systemic iron overload.

Together with albumin and many other plasma proteins, also a fraction of plasma–transferrin is filtered through the glomerulus, and re-absorbed from the primary urine into kidney epithelial cells. TfR1 is found apically on kidney epithelium [10] (and Fig 2) and takes up holo-Tf, in an iron regulated manner. It is strongly down-regulated during PIO (Fig 3), thus it is not likely for the Tf-TfR1 system to be the main route for Tf and iron uptake from primary urine of iron overloaded mice. In contrast, upregulation of megalin/cubilin during PIO suggests that Tf-iron is mainly reabsorbed by cubilin under these conditions, which explains why Tf reabsorption through the kidney is not limited during systemic iron overload and none of this protein is found in urine. In a rat kidney cell model, megalin regulation by iron and the functional competition between TfR1 and megalin was suggested [46]. Recently also cubilin upregulation was observed in a mouse-model for hemolytic anemia and this was accompanied by increased function of the megalin/cubilin complex [47]. A patient with two mutations in the megalin gene, which led to a mostly intracellular location of megalin and absence of membrane megalin, had elevated urinary levels of cubilin and type 3 carbonic anhydrase due to shedding of these proteins [48]. Thus cubilin, which has no trans-membrane domain and depends on megalin for its membrane location and internalization, may possibly be stabilized by megalin during PIO.

Once in the epithelial cells, iron needs to be transported across the cells. We have previously shown a possible role for ferritin in both intra- and intercellular iron trafficking in the Sertoli cells of the testis [49]. There we suggested, that iron that is taken up apically by Sertoli cells may traffic within ferritin to the basolateral pole of these epithelial cells, where ferritin is secreted in a regulated way. In macrophages, much intracellular ferritin is found in membrane bound vesicles of the endo-lysosomal system and manipulation of the endo/lysosomal trafficking machinery affects ferritin secretion [50]. The distinct distribution of ferritin in renal epithelial cells of iron overloaded mice and of mice suffering from a functional iron deficiency (Irp2-/- mice) suggests that iron status regulates the trafficking of ferritin containing vesicles, which are located near the apical membrane, in iron deficient cells, and are dispersed throughout the cell and near the basolateral membrane in iron overloaded cells. Similarly, transferrin-iron has been shown to be involved in the regulation of endosomal trafficking in erythroid cells [51, 52]. This finding further suggests that ferritin may be secreted basolaterally and contribute to the iron flux through renal epithelial cells. Yet, in a cell-model of proximal tubule cells, no ferritin was detected in the basolateral compartment, in the first 4–7 hours of apical iron exposure [23].

Ferrous iron can be exported through FPN, which is located basolaterally in kidney epithelium [27]. In our hands, FPN was strongly reduced in the spleen of the PIO mice and slightly reduced in Irp2-/- mice, as described [53]. Yet in the kidneys FPN levels were not reduced by PIO, suggesting that the different regulatory forces acting on FPN [30, 54] are balancing it to remain unchanged in iron overload, in the kidney. The slight elevation of FPN in the kidneys of Irp2-/- mice further supports the notion that IRPs contribute to FPN regulation in the kidney. This implies that iron may not only be efficiently imported to kidney epithelial cells during iron overload but it may also be efficiently exported to the interstitium and the blood. Interestingly, in response to dietary iron overload, cortical epithelial cells were the major sites of iron accumulation, which stood in contrast to the pattern of iron accumulation in PIO (Fig 6). This discrepancy may origin in a different ratio of Tf-bound iron and NTBI reaching the primary urine in the two ways of iron overload, which will affect the site of reabsorption along the tubule and subsequent handling of iron. In addition, we speculated that different hepcidin levels may offer an explanation for the differential iron distribution. However liver hepcidin levels increased about four-fold in both, dietary iron overload [55] and PIO (control 1.22±0.8; PIO 4.61±0.67; p<0.005 n = 3). It remains possible that in response to dietary iron administration, renal hepcidin production may increase more than in response to PIO and that this plays a role in the kidney iron distribution [56]. More research may clarify these hypotheses.

Iron chemistry is dominated by the inter-conversion of ferrous and ferric iron [57], which are maintained at equilibrium. The low oxygen conditions in the renal medulla (1.3–2.6% O_2_) support an iron homeostasis with slightly higher concentrations of ferrous iron in solution than in the well-oxygenated cortex (6.6% O_2_) [58, 59]. Thus, ferrous iron transport may be facilitated across cellular plasma membranes in the medulla, which may permit the medulla to maintain a highly dynamic iron pool that is not used for long-term iron storage.

We can think of two biological functions for efficient iron re-absorption of the kidney also when systemic iron is high. 1) Erythropoiesis is the most iron consuming process in the body and is regulated by erythropoietin (epo), made in the kidney interstitium [60]. Epo is regulated mainly by HIF, which senses both oxygen and iron, thus integrating the systemic need for red blood cells and the systemic ability to make them. Hence, if the iron flux through the kidney represents systemic iron stores, rather than responds to- and regulates these stores, important information is convened to the regulatory system of erythropoiesis. 2) There is a tough competition for iron acquisition between host and pathogens [61]. The iron mediated up-regulation of the multi-ligand receptor complex megalin/cubilin facilitates not only the efficient re-absorption of Tf protein and its bound iron but also uptake of iron bound to other molecules. Thus, it may be part of a mechanism that prevents bacteria causing urinary tract infections [62] to thrive during systemic iron overload. On the other hand, with the megalin/cubilin complex being able to bind and internalize many other and potentially harmful molecules including carcinogens and drugs its upregulation may contribute to the toxicity of iron overload [48].

Taken together, we have evidence that during PIO, iron is transported efficiently across the epithelial barrier. TfR1 levels are low, but cubilin levels are high and the cubilin-megalin heterodimer likely plays a major role in iron transport from the primary urine back to the body. Ferritin is distributed throughout the epithelial cells and does not accumulate at the apical brush-border, suggesting that it may contribute to intra- and inter-cellular iron trafficking. FPN is not down-regulated by the high iron conditions and thus may export iron efficiently from the basolateral epithelium into the renal interstitium and also from interstitial macrophages. We suggest that the highly expressed cubilin-megalin complex mediates Tf-iron re-absorption during PIO both in the cortex and the medulla, where excess iron is stored predominantly in interstitial medullar macrophages. The strategy of shifting a significant part of iron re-absorption to the medulla during PIO may accelerate renal iron flux. In conclusion, we suggest that iron transport through the kidney epithelium is unique in its regulation, re-absorbing iron even when systemic iron is high. This may protect the host from uropathogenic bacteria and provide erythropoietin producing cells with important information on body iron stores.
